# Inhibition of germinal vesicle breakdown using IBMX increases microRNA-21 in the porcine oocyte

**DOI:** 10.1186/s12958-020-00603-1

**Published:** 2020-05-11

**Authors:** Benjamin J. Hale, Yunsheng Li, Malavika K. Adur, Jason W. Ross

**Affiliations:** Department of Animal Science, 2356 Kildee Hall, Iowa State University, Ames, IA 50011 USA

**Keywords:** Oocyte, Germinal vesicle breakdown, microRNA, MIR21

## Abstract

**Background:**

Germinal vesicle breakdown (GVBD) occurs during oocyte meiotic maturation, a period when transcriptional processes are virtually inactive. Thus, the maturing oocyte is reliant on processes such as post-transcriptional gene regulation (PTGR) to regulate the mRNA and protein repertoire. MicroRNA (miRNA) are a class of functional small RNA that target mRNA to affect their abundance and translational efficiency. Of particular importance is miRNA-21 (MIR21) due to its role in regulating programmed cell death 4 (PDCD4). The objective of this study was to characterize the abundance and regulation of MIR21 in relation to GVBD.

**Methods:**

Oocytes were collected from aspirated porcine tertiary follicles. Relative abundance of mature MIR21 was quantified at 0, 8, 16, 24, 32, and 42 h of in vitro (IVM) with or without treatment with 3-isobutyl-1-methylxanthine (IBMX).

**Results:**

IBMX increased abundance of MIR21 at 24 h approximately 30-fold compared to control oocytes (*P* < 0.05), and the induced increase in MIR21 abundance at 24 h was concomitant with premature depletion of PDCD4 protein abundance. To characterize the effect of artificially increasing MIR21 on oocyte competence without inhibiting GVBD, a MIR21 mimic, scrambled microRNA negative control, or nuclease free water was micro-injected into denuded oocytes at 21 h of IVM. The maturation rate of oocytes injected with synthetic MIR21 (63.0 ± 7.5%) was higher than oocytes injected with negative controls (*P* < 0.05).

**Conclusions:**

Inhibition of nuclear meiotic maturation via IBMX significantly increased MIR21 and decreased its target, PDCD4. Injection of a MIR21 mimic increased oocyte maturation rate. Our results indicate MIR21 is active and important during meiotic maturation of the oocyte.

## Background

Maturation of the mammalian oocyte is a complex process involving internal checkpoints and bidirectional communication with the surrounding cumulus cells. The oocyte must maintain arrest at the diplotene stage until meiotic resumption occurs. Oocyte arrest at the diplotene stage is maintained in part through the activity of phosphodiesterase enzymes that catalyze the hydrolysis of cyclic adenosine monophosphate (cAMP) into AMP [1, 2]. Inhibition of phosphodiesterase activity induces oocyte meiotic maturation as activation of G-protein coupled receptors (GPCR) on the plasma membrane activates adenylate cyclase [3, 4]. The regulatory role of cAMP in meiotic arrest is further illustrated by studies using selective phosphodiesterase 3A (PDE3A) inhibitors to block nuclear maturation [5–8].

Following germinal vesicle break down (GVBD) the oocyte is transcriptionally quiescent until fertilization and activation of the embryonic genome, occurring at the four-cell stage of development in the pig [9]. The inability to transcribe mRNA during this stage of development and the probable necessity for post-transcriptional gene regulation (PTGR) suggests an important role for non-coding RNA in the maturing oocyte. MicroRNA (miRNA) are abundantly present in oocytes of multiple species during various stages of development [10–15], but it remains unclear how GVBD affects miRNA biogenesis.

Biogenesis of miRNA occurs in a step-wise manner starting with a double-stranded precursor [16–18]. The DGCR8/Drosha microprocessing complex first cleaves this double-stranded precursor, called the primary miRNA (pri-miRNA), creating the second precursor form, the pre-miRNA [18, 19]. In somatic cells, the pre-miRNA is exported out of the nucleus by Exportin-5 (EXPO5) [20, 21], where it is further processed by Dicer into the mature form [22, 23]. The single-stranded mature miRNA can then associate with an Argonaute protein, which mediates its function of mRNA regulation through direct mRNA target cleavage or interactions with associated RNA-induced silencing complex (RISC) proteins [24].

Of particular interest is understanding the role of non-coding RNA in regulating meiotic checkpoints in the oocyte, particularly that of microRNA-21 (MIR21). The role for MIR21 in malignancy was first reported by Chan et al. [25], and is now known to be ubiquitously expressed in cancer cells [26] as MIR21 targets and inhibits pro-apoptotic proteins, programmed cell death 4 (PDCD4) and phosphatase and tensin homolog (PTEN) [27, 28]. Pri-MIR21 can be transcribed in coordination with the expression of vacuole membrane protein 1 (VMP1; formerly TMEM49), as MIR21 resides in the tenth intron of VMP1, although it appears MIR21 can be regulated independently as well due to a co-localized promoter within the VMP1 intron [29].

We have previously demonstrated an elevated abundance of MIR21 in metaphase II-arrested (MII) oocytes compared to immature oocytes and that inhibiting MIR21 during oocyte maturation decreases the number of oocytes capable of reaching the MII arrest [30]. Based on the apparent role of MIR21 in maintaining meiotic competence in the pig oocyte and that the oocyte undergoes a dramatic change in chromatin organization during meiotic maturation, we hypothesized that inhibition of GVBD would affect the abundance of mature MIR21 in the oocyte. MiRNA biogenesis is dependent on the transport of pre-miRNA out of the nucleus. We therefore predicted that the occurrence of GVBD in the oocyte potentially releases precursor miRNA out into the ooplasm and allows for further processing by Dicer to generate a mature miRNA. Our data suggest that this is likely not the case. The comparison of MIR21 abundance throughout in vitro maturation (IVM) with or without inhibition of GVBD suggests that miRNA biogenesis occurs before GVBD.

## Methods

### In vitro maturation

Sow ovaries were obtained from a local abattoir for isolation of cumulus-oocyte-complexes (COCs) for in vitro maturation (IVM) as previously described [14, 31]. Briefly, follicles (2–4 mm) were aspirated and COCs were collected and washed in TL-Hepes with 0.1% polyvinyl alcohol (PVA). COCs were cultured in maturation media (Tissue Culture Media 199 (TCM-199)) containing 0.57 mM L-cysteine, follicle stimulating hormone (0.5 μg/mL), luteinizing hormone (0.5 μg/mL), and epidermal growth factor (10 ng/mL) for at most 42 h at 38.5 °C in 5% CO_2_. Prior to IVM, an aliquot of germinal vesicle-intact (GV) stage oocytes for each replication were randomly selected from the COC pool. GV stage oocytes used for analysis were stripped of cumulus cells via vortex (5 to 7 min) in 1% hyaluronidase in TL-Hepes-PVA. COCs that were collected at 8, 16, 24, 32, or 42 h of IVM were stripped of cumulus cells in the same manner.

### Utilization of IBMX to inhibit germinal vesicle breakdown

Pools of approximately 70 COCs were cultured under normal IVM conditions (as previously described) with media containing 1.0 mM IBMX or DMSO vehicle control. The concentration of 1.0 mM IBMX was selected based on a titration of 0.5 mM, 1.0 mM, or 2.0 mM of IBMX, for which the range was selected based on previous studies [32]. Oocytes were stripped of cumulus cells as described above before initial culture (0 h) or 8, 16, 24, 32, or 42 h past initial culture. Four technical replicates were performed for each time course and treatment. Denuded oocytes were collected based on the presence of an extruded polar body and morphologically healthy appearance, based on an appropriate zona pellucida integrity, the absence of ooplasm degradation, and proper perivitelline space. For each time point, oocytes were randomly divided into two pools of 25; one pool was flash frozen for RNA extraction, and the other was fixed in 4% paraformaldehyde (PFA)/PBS at 4 °C for 24 h, as in previous studies [30]. Fixed oocytes were washed and mounted on slides with VectaShield mounting media containing DAPI stain (Vector Laboratories®, Burlingame, CA). Chromatin configurations were viewed for each of the 25 oocytes from vehicle control or IBMX containing culture media. Mounted oocytes were scored as either germinal vesicle-intact (GV) or having undergone or undergoing GVBD. The percentage of either GV or GVBD was calculated for each time point.

### Quantification of MIR21 abundance

Oocytes were collected and denuded of cumulus cells at the time points described above, with four technical replicates. Oocytes from each time point and treatment were collected in pools of exactly 25 oocytes with minimal amount of TL-Hepes/PVA. As previously established, a precise number of denuded oocytes were used per reaction to avoid the introduction of additional variation associated with reference genes (14,30). TaqMan™ Gene Expression Cells-to-Ct™ Kit (Applied Biosystems, Carlsbad, CA) was used to lyse oocytes and prepare samples for quantitative polymerase chain reaction (qRT-PCR). Lysis solution and DNase from the Cells-to-C_T_**™** kit (Invitrogen**™** Ambion**™**) were added to each pool at 4.95 and 0.05 μL, respectively, and incubated at room temperature for 5 min. Stop solution (0.5 μL) was added and the samples were incubated for an additional two minutes.

MIR21 was quantified using a TaqMan® MicroRNA Reverse Transcription kit (Applied Biosystems, Carlsbad, CA) for the reverse transcription (RT) reaction and the primers and probe used were TaqMan® MicroRNA Assay for hsa-MIR21 (Applied Biosystems, Carlsbad, CA) as per manufacturer’s recommendations. The RT reaction volume was 20 μL consisting of 13 μL master mix, 3 μL primers, and 4 μL sample lysate. Reverse transcription conditions were 16 °C for 30 min, 42 °C for 30 min and 85 °C for 5 min. The final volume for all quantitative RT-PCR reactions was 20 μL, which included 1.33 μL of the RT product, 1 μL TaqMan MicroRNA Assay (20x), 10 μL TaqMan 2x Universal PCR Master Mix, and 7.67 nuclease free water. The thermal cycling conditions for the TaqMan MicroRNA RT-PCR were 95 °C for 10 min, followed by 45 cycles of 95 °C for 15 s and 60 °C for 60 s. Fluorescent data acquisition was during the 60 °C extension step.

### Injection of MIR21 mimic during IVM

After approximately 21 h of IVM, COCs were denuded in 0.5 mg/mL hyaluronidase with gentle vortexing. The oocytes were washed in manipulation media after cumulus cells were removed, and oocytes of good or excellent morphology were selected for injection. Injection occurred after 21 h of IVM because injection of porcine GV-intact oocytes is more likely to be detrimental and cause oocyte demise (data not shown) and to mimic the artificial increase of MIR21 abundance due to IBMX between 16 and 24 h of IVM. Denuded oocytes were injected using 0.7 μm diameter Femptotip®II tips (Eppendorf**™**, Hamburg, Germany) with 10 pL of nuclease-free water, 25 nM microRNA mirVana**™** negative control (Ambion**™**, Connecticut, USA), or 25 nM mature 5′ MIR21 mimic (Integrated DNA Technologies, New Jersey, USA). Injected oocytes were cultured for an additional 21 h. After a total of 42 h of IVM, oocytes were assessed for their ability to achieve MII arrest based on the presence or absence of a polar body and a morphologically healthy appearance. Also at this time, 50 MII-arrested oocytes per treatment were pooled and flash frozen in liquid nitrogen. Oocytes were flash-frozen in liquid nitrogen and stored at − 80 °C for SDS-PAGE separation and Western blotting. Four technical replicates using 50 oocytes for each of the three treatments was performed.

## Western blot analysis

Pools of 100 denuded oocytes within a replication were collected as described above for 0, 24 and 42 h past initial culture with either DMSO loading control or 1.0 mM IBMX, and stored at − 80 °C until used for Western blot analysis. Oocyte pools were lysed in 2.5 μL of 10% SDS (total sample volume 12.5 μL) at 95 °C for 4 min followed by 1 min on ice and centrifugation at 1000 rpm for 1 min at room temperature. Samples were then loaded into a 4–20% Tris glycine gel (Lonza PAGEr® Gold Precast Gels). The BioRad Mini PROTEAN Tetra System was used to separate proteins at 60 V for 30 min followed by 120 V for 90 min. The proteins were transferred to a nitrocellulose membrane for 1 h at 100 V at 4 °C. Membrane blocking was conducted using 5% milk in PBST (PBS with 0.5% Tween 20) for 1 h at room temperature. A rabbit anti-PDCD4 monoclonal antibody (Abcam, ab79405) was added (1:1000 dilution) to the membrane in 0.5% milk in PBST overnight at 4 °C. A negative control membrane lacking primary antibody was included to control for secondary antibody nonspecific binding. Following primary antibody incubation, the membranes were washed with PBST three times at room temperature for 10 min. Donkey anti-rabbit IgG (Amersham™ ECL™ NA934) was incubated (1:2000) with the membrane for 1 h at room temperature. The membrane was washed three times for 10 min each at room temperature. Horseradish peroxidase substrate (Millipore, Billerica, MA) was added to the membrane for 1 min in the dark. The membrane was exposed to x-ray film and developed for visualization. Average pixel intensity for the protein corresponding to 52 kDa for PDCD4 molecular weight was conducted using Image Studio™ Lite (Li-Core®). Ponceau S stain was used as a loading control to normalize total protein transferred to membrane.

### Statistical analysis

Statistical analysis of oocyte maturation rate, qRT-PCR relative RNA abundance, and Western blot data was conducted using PROC MIXED in SAS® (Carry, NC), where a standard student t-test was used to compare statistical differences. Statistical significance was determined when *P* values were less than or equal to 0.05. Analysis of oocyte maturation rate after injection utilized repetition number as a covariate to account for differences between repetitions.

## Results

### IBMX inhibition of oocyte nuclear maturation increases MIR21 abundance

**Fig. 1:**
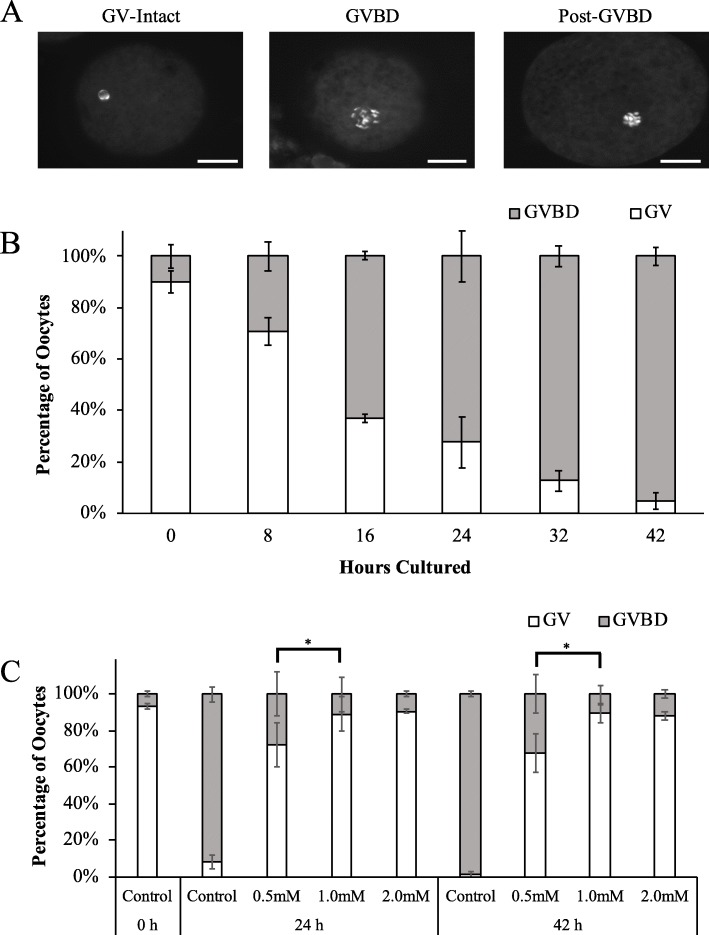
Quantification of Germinal Vesicle Breakdown during In Vitro Maturation. Cumulus-oocyte-complexes (COCs) were aspirated from 2 to 4 mm follicles and allowed to undergo in vitro maturation (IVM). **a** Representative images indicating how oocytes were scored as containing an intact germinal vesicle (GV) or undergoing or have undergone germinal vesicle breakdown (GVBD). White scale bars represent 25 μm. **b** COCs were collected from IVM at 8-h intervals, denuded, fixed, and the chromatin was stained with DAPI. GVBD increased rapidly from 8 to 24 h of IVM, where by 24 h of IVM approximately 75% of the oocytes collected were scored as GVBD. **c** Vehicle control, 0.5 mM, 1.0 mM, or 2.0 mM IBMX was added to IVM media, and oocytes were collected at 0, 24, and 42 h of IVM to be scored at either GV-intact or having undergone GVBD. All three concentrations of IBMX inhibited GVBD. Asterisks denote significant difference (*P* < 0.05)

To compare the increase of MIR21 abundance directly to the percentage of oocytes undergoing GVBD, oocytes were scored as GV-intact, GVBD, or post GVBD at 8-h intervals during IVM (Fig. 1a and b). There was a marked increase in the occurrence of GVBD between 0 and 16 h of IVM, by which approximately 63% of the oocytes had undergone GVBD (Fig. 1b).

To characterize the inhibition of GVBD under different concentrations of IBMX, vehicle control, 0.5 mM, 1.0 mM, or 2.0 mM IBMX was added to IVM media, and oocytes were collected at 0, 24, and 42 h of IVM to be scored as either GV-intact or having undergone GVBD. As expected, all three concentrations of IBMX in IVM media inhibited GVBD (Fig. 1c).

Compared to the percentage of GV-intact oocytes after IVM in the presence of 0.5 mM IBMX (68.0% ± 12.9%), there was a decreased percentage of oocytes with an intact GV after 42 h of IVM with 1.0 mM IBMX (89.3% ± 5.9%; *P* = 0.047) and a tendency for decreased GV-intact oocytes with 2.0 mM IBMX (88.0% ± 2.8%; *P* = 0.06). The inclusion of 2.0 mM IBMX did not further decrease the number of GV-intact oocytes, compared to oocytes that underwent IVM in the presence of 1.0 mM IBMX (*P* = 0.894). For all further experiments 1.0 mM IBMX was used.

**Fig. 2:**
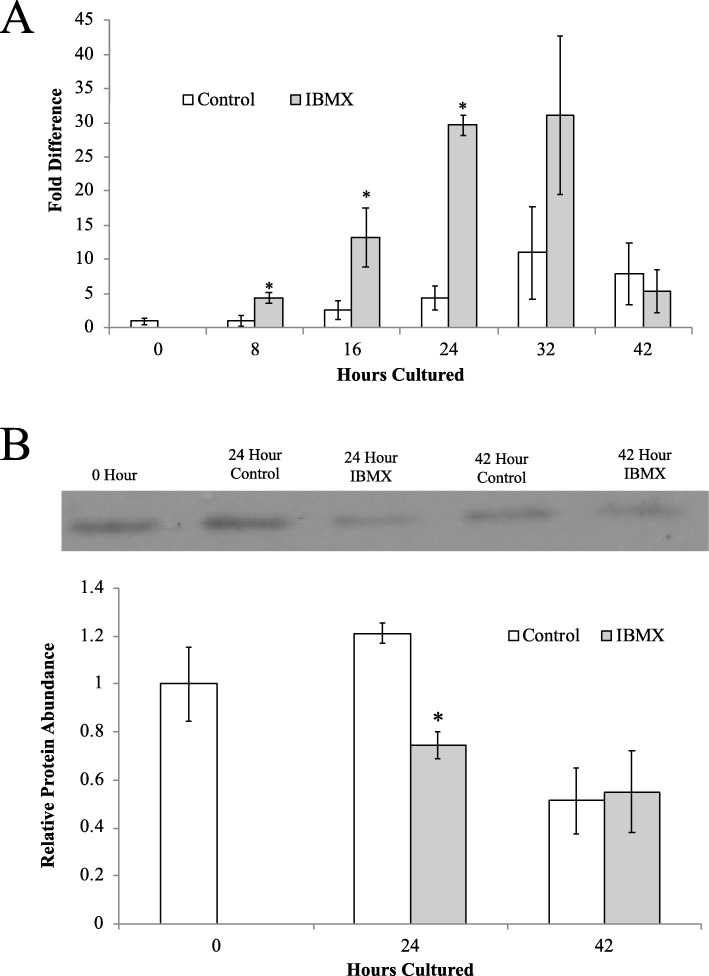
Inhibition of GVBD Increased MIR21 Abundance. Collected COCs underwent IVM in the presence of a vehicle control or 1.0 mM IBMX, and then collected and denuded at 8 h intervals. The oocytes that underwent IVM in the presence of IBMX had approximately 30-fold greater MIR21 abundance at 24 h of IVM compared to control oocytes (**a**). This increase of MIR21 at 21 h of IVM was temporally associated with decreased PDCD4 abundance (**b**) suggesting the increased MIR21 due to IBMX prevented GVBD is biologically active within the oocyte. Asterisks denote significant difference (*P* < 0.05)

To assess potential mechanisms contributing to the increase of MIR21 occurring in the maturing oocyte, IBMX was used to inhibit the nuclear maturation of oocytes in IVM conditions by preventing GVBD. The IBMX supplemented IVM media contained the hormonal signals and nutrients necessary for cytoplasmic maturation. In oocytes under control IVM conditions, MIR21 abundance increased during IVM as we have shown previously [30]. The inclusion of 1.0 mM IBMX increased the abundance of MIR21 (29.6 ± 1.5-fold) by 24 h of IVM compared to DMSO loading control (4.5 ± 1.8-fold; *P* < 0.01; Fig. 2a). There was a subsequent decrease in PDCD4 protein (0.75 ± 0.05 relative band intensity) in oocytes under IBMX inhibition at 24 h compared to control (1.2 ± 0.04 relative band intensity; *P* = 0.03; Fig. 2b). This suggests that IBMX inhibition of GVBD increased the abundance of MIR21 in the oocyte hastening the MIR21-driven decrease in PDCD4 abundance.

### Injection of MIR21 at 21 h of IVM increases oocyte maturation

After 21 h of IVM, oocytes were denuded and injected with either a MIR21 mimic, microRNA scrambled negative control, or nuclease-free water and allowed to continue IVM until a total of 42 h. The IVM oocytes were injected at 21 h to mimic the increase in MIR21 abundance observed in control oocytes by 24 h of IVM (Fig. 2a). The time point of 21 h was chosen because it was prior to the observed increased in MIR21, so that it can be utilized for target mRNA inhibition at or earlier than 24 h of IVM. Injections were not done earlier than 21 h of IVM as it was a concern that exogenous single-stranded RNA would be degraded if unutilized [33]; though degradation of exogenous RNA was not specifically quantified in this study.

**Fig. 3:**
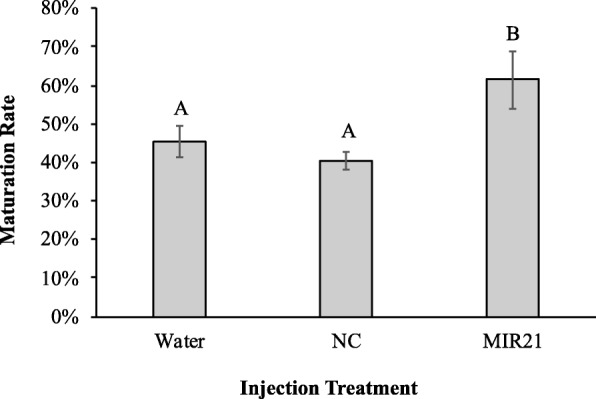
Injection of MIR21 Increases Maturation Rate. Collected COCs underwent IVM for 21 h, when they were then denuded and injected with either scrambled miRNA negative control (NC), water loading control (water), or synthetic mature MIR21. The oocytes were then placed back into IVM media until a total of 42 h. Injection of MIR21 increased maturation rate compared to oocytes injected with either control. Superscripts denote significant difference (*P* < 0.05)

Maturation rate for each treatment was scored via the presence of a morphologically healthy metaphase II arrested oocyte containing an extruded polar body. The injection of MIR21 increased the maturation rate of oocytes (61.6% ± 0.75%) compared to the injection of either scrambled microRNA (40.3% ± 0.02%; *P* = 0.004) or water (45.4% ± 0.4%; *P* = 0.015; Fig. 3). The oocyte maturation rate was not different between the two negative controls (*P* = 0.15; Fig. 3). These results indicate that MIR21 is active and affects meiotic maturation in the porcine oocyte.

## Discussion

MIR21 is an anti-apoptotic factor that is ubiquitously found in cancer cells [26]. This is in part due to its ability to target and suppress pro-apoptotic proteins, including PDCD4 and PTEN [27]. Predictions using TargetScan v6.2 suggest there are currently 382 predicted MIR21 targets identified in the human genome [34]. Besides targets regulating apoptosis, MIR21 also has the potential to regulate pathways associated with cell cycle arrest, suppression of tumor growth, and chromosome assembly [26, 35]. We have previously discovered increased MIR21 abundance in MII porcine oocyte compared to immature oocytes [14], and that inhibition of MIR21 function in the maturing oocyte increased PDCD4 abundance and decreased developmental competence [30]. Furthermore, increased MIR21 in the maturing oocyte potentially originates from the oocyte and not solely from the surrounding cumulus cells [30]. Due to the important role of MIR21 in regulating cell cycle decisions in somatic cells, and its abundance in the maturing oocyte, it is likely that MIR21 is involved in meiotic maturation in the female germ cell.

In this project, IBMX, a phosphodiesterase inhibitor, was used to prevent GVBD during IVM of oocytes to test the hypothesis that GVBD affects MIR21 abundance. In somatic cells, miRNA are first transcribed as primary miRNA (pri-miRNA), which form secondary RNA structures producing 60–75 nucleotide (nt) hairpins [16, 36]. The canonical miRNA hairpins are recognized by DGCR8 (DiGeorge syndrome critical region 8), which directs the RNase III enzyme Drosha to cleave the base of the hairpin resulting in a pre-miRNA [17–19]. This pre-miRNA structure, composed of a hairpin with a 3′ overhang is exported out of the nucleus via EXPO5 [20, 21] and then further processed by Dicer in complex with transactivating response element RNA-binding protein (TRBP), resulting in a functionally mature miRNA [16, 23, 37–39].

During maturation, the oocyte experiences a period following GVBD where the nuclear material is no longer encapsulated by the nucleolus, potentially affecting microRNA biogenesis due to the reduced requirement of EXPO5 to facilitate pre-miRNA out of the nucleus. Further supporting this concept is that EXPO5 mRNA is relatively lowly expressed in the maturing pig oocyte relative to the other molecules known to affect canonical miRNA biogenesis [40]. Based upon this knowledge, we developed our hypothesis that inhibition of GVBD could do one of two things: 1) decrease the abundance of mature MIR21, since immature MIR21 would be sequestered within the GV, or 2) increase the abundance of mature MIR21, since the oocyte would have a prolonged ability to transcribe immature MIR21. To decipher this, nuclear maturation of the oocytes was inhibited through IBMX and the abundance of MIR21 was determined.

IBMX is a nonspecific phosphodiesterase inhibitor which inhibits adenylate cyclase within the oocyte [41]. This prolongs the elevated levels of cAMP in the oocyte and obstructs oocyte nuclear maturation [42, 43]. Though the process of GVBD and the inhibition of GVBD via IBMX has been well characterized in the pig oocyte during IVM [32, 44, 45], the approach used herein enabled the temporal characterization of MIR21 changes in abundance and GVBD at 8-h intervals. It is important to note that we did not characterize how IBMX affects the biogenesis of MIR21, but instead focused on quantifying the abundance of mature MIR21 and its target, PDCD4, during specific time points in GVBD.

In control oocytes, mature MIR21 was elevated by 24 h of IVM and remained elevated until 42 h. Interestingly, oocytes matured in the presence of IBMX had much greater MIR21 than control oocytes at 24 h of IVM. This 24 h time point corresponds to when approximately 75% of control oocytes undergo GVBD which is synonymous with transcriptional quiescence [46]. The greater abundance of MIR21 in IBMX oocytes compared to control oocytes at 24 h of IVM could be explained by the fact that 75% more oocytes in the IBMX treatment are maintaining the ability to produce nascent RNA. This increase of MIR21 abundance between 0 and 24 h of IVM could be important for maintaining oocyte viability during maturation. The increase of MIR21 in oocytes matured for 24 h in the presence of IBMX was followed by a numerical decrease in MIR21 abundance at 42 h of IVM in the presence of IBMX. This is potentially explained by the artificial increase of MIR21 resulting in a negative feedback loop, as in other cells [27].

We further aimed to characterize the importance of the MIR21 increase from 0 to 24 h of IVM. If this increase is paramount to maintaining viability, then artificially increasing MIR21 before 24 h of IVM without inhibiting GVBD could increase the maturation rate of IVM oocytes. To characterize the effect of artificially increasing MIR21 on oocyte competence without inhibiting GVBD, a synthesized MIR21 mimic or controls, were micro-injected into the oocyte at 21 h of IVM. In comparison to the control oocytes, the maturation rate of oocytes injected with MIR21 mimic was increased. This further indicates the biological machinery available for MIR21-mediated PTGR in the oocyte, as we predicted based on previous studies [30]. These results suggest that MIR21 is active within the porcine oocyte and that it has a role in regulating oocyte competence during maturation.

By taking a targeted approach, we have previously shown that inhibition of a specific miRNA, MIR21, in the oocyte decreases oocyte maturation [30]. Here we characterize the increase of MIR21 in relation to GVBD, and that artificially increasing MIR21 affects maturation rate. These cumulative results suggest a functional role of miRNA in porcine oocyte maturation. Using conditional knock-out models, it has been suggested that miRNA function is repressed during oocyte maturation in rodents [47] and our findings suggest some potential species differences with respect to miRNA function.

The cumulative results of this study fortify the argument that miRNAs are active and important for meiotic maturation of the female germ cell. After GVBD the oocyte is transcriptionally quiescent until after fertilization and activation of the embryonic genome [48]. The level of nascent mRNA produced after GVBD is almost nonexistent, and the oocyte relies heavily on PTGR and other mechanisms to regulate the dynamic processes involved in chromosomal migration as well as react to cellular or environmental stress. MiRNA is a potential mechanism the oocyte utilizes to enact PTGR.

Morphological markers for the integrity of immature oocytes are imperfect at predicting whether the oocyte contains the necessary material to mature and undergo fertilization [49]. MiRNA potentially represent more informative markers of oocyte viability, and increasing the understanding of the components necessary for oocyte viability is necessary for improving the efficiency of assisted reproductive techniques [50, 51].

## Conclusions

The present study shows that inhibiting porcine oocyte GVBD artificially increases MIR21 abundance, and the results presented here suggest a functional role of MIR21 in porcine oocyte maturation. This has clinical implications as functional RNAs represent potential markers of oocyte quality.

## Data Availability

Not applicable.

## Abbreviations

GVBD: Germinal vesicle breakdown
miRNA: MicroRNA
MIR21: MicroRNA-21
PDCD4: Programmed cell death 4
IVM: In vitro maturation
IBMX: 3-isobutyl-1-methyxanthine
PTGR.: Post-transcriptional gene regulation
